# MXD3 regulation of DAOY cell proliferation dictated by time course of activation

**DOI:** 10.1186/1471-2121-15-30

**Published:** 2014-07-23

**Authors:** Tin Ngo, Gustavo A Barisone, Kit S Lam, Elva Dίaz

**Affiliations:** 1 Department of Pharmacology, UC Davis School of Medicine, 451 Health Sciences Drive, 3503 GBSF, Davis, CA 95616, USA; 2 Department of Biochemistry and Molecular Medicine, University of California Davis School of Medicine, Davis, CA, USA

**Keywords:** MXD3, Proliferation, Medulloblastoma, DAOY

## Abstract

**Background:**

MXD3 is a basic-helix-loop-helix-leucine-zipper transcription factor involved in cellular proliferation. In previous studies we demonstrated that knock-down of MXD3 in the human medulloblastoma cell line DAOY resulted in decreased proliferation. Surprisingly, overexpression of MXD3 in DAOY cells also decreased proliferation and increased cell death, suggesting that persistent expression of MXD3 triggers an apoptotic response, perhaps as a fail-safe mechanism. To investigate this apparent paradox in detail we developed a tamoxifen inducible system to analyze the temporal effects of MXD3 in the proliferation and transcriptional response of DAOY cells upon acute induction compared with long-term expression of MXD3.

**Results:**

We find that acute induction of MXD3 initially promotes cell cycle progression as assessed by a transient increase in bromodeoxyuridine incorporation. However, persistent induction of MXD3 ultimately results in decreased proliferation based on cell counts. Finally, with microarray expression profiling and gene ontology analysis we identify several major pathways enriched in response to acute (immune response, apoptosis, cell cycle) versus persistent (cell adhesion) MXD3 activation.

**Conclusions:**

In this study, we demonstrate that acute MXD3 activation results in a transient increase in cell proliferation while persistent activation of MXD3 eventually results in an overall decrease in cell number, suggesting that the time course of MXD3 expression dictates the cellular outcome. Microarray expression profiling and gene ontology analysis indicate that MXD3 regulates distinct genes and pathways upon acute induction compared with persistent expression, suggesting that the cellular outcome is specified by changes in MXD3 transcriptional program in a time-dependent manner.

## Background

Medulloblastoma, the most common brain tumor in children [1], develops due to uncontrolled proliferation of cerebellar granule neuron precursors (GNPs) [2]. A large body of literature exists regarding the molecular mechanisms of medulloblastoma formation and progression. Thus far, four subtypes of medulloblastomas have been identified including the Wnt and Sonic hedgehog (Shh) subgroups [3,4]. Medulloblastomas of the Shh subgroup have mutations in upstream components of the Shh pathway, including the receptors PTCH and SMO [4]. PTCH, when bound by Shh, relieves its inhibition of SMO [5] which then initiates a complex cascade of events leading to cell cycle progression. One example of an established mechanism for Shh pathway-dependent cell cycle progression is through the upregulation of cyclins by the proto-oncogene MYCN [6]. Mutation of downstream targets of Shh such as GLI1, GLI2 [7], and MYCN is a characteristic of medulloblastomas within the Shh subtype [4]. However, the molecular mechanisms behind medulloblastoma formation and progression are not completely understood. Indeed, recent evidence suggests that a subset of cerebellar granule neurons originate not from GNPs but from a population of Nestin-expressing progenitors (NEPs) in the deep external germinal layer and that these NEPs are more susceptible to Shh-dependent tumor formation [8].

Previously, our lab identified the transcription factor MXD3 as a critical regulator of GNP proliferation during normal cerebellar development as a downstream component of the Shh pathway [9]. Interestingly, we found that MXD3 is overexpressed in tumor tissue from the PTCH deficient heterozygote mouse model of medulloblastoma [9]. Moreover, recently we showed that MXD3 is upregulated in human medulloblastomas and is required for the proliferation of the human medulloblastoma cell line DAOY [10]. These results suggest a role for MXD3 in medulloblastoma in humans. The DAOY cell line was established from a medulloblastoma tumor mass obtained from a 4 year old patient [11]. Tissue from this tumor had evidence of both neural and glial differentiation; however, these characteristics were lost during the establishment of DAOY as a cancer cell line [11].

MXD3 is a basic-helix-loop-helix-leucine-zipper (bHLHZ) transcription factor that is part of the MYC/MAX/MXD transcriptional network [12]. Within this network, MYC and MXD family members compete with each other for MAX heterodimerization to invoke opposing transcriptional regulation of target genes [13,14]. Specifically, MYC and MAX heterodimers recruit transcriptional activators [15] while MXD and MAX heterodimers recruit transcriptional repressors [13,16,17]. MYC family members have been shown to promote while MXD family members have been shown to repress cell cycle progression [18]. MXD3, however, is an atypical member of the MXD family as it has been found to be expressed during the S-phase of the cell cycle [9,19,20] while other MXD family members are expressed in differentiated cells [14]. Knockdown of MXD3 leads to a reduction in cell number suggesting that MXD3 is required for cell cycle progression [9,10]. On the other hand, overexpression of MXD3 is sufficient to promote proliferation in mouse cerebellar GNPs [9]. Consistent with these results, overexpression of MXD3 negatively regulates differentiation in mouse B cells derived from the spleen [21]. *Persistent* overexpression of MXD3, however, in mouse GNPs and in human medulloblastoma cells results in decreased proliferation due to the activation of apoptosis [9,10].

To characterize MXD3 overexpression in a time dependent manner, we engineered the DAOY cell line to express stably a fusion protein between the truncated Estrogen Receptor and MXD3 (ER-MXD3). In contrast to endogenous MXD3, which is localized to the nucleus [22], under baseline conditions the ER-MXD3 fusion protein is enriched in the cytoplasm. Upon treatment of 4-hydroxytamoxifen (4-OHT) the ER-MXD3 fusion protein translocates into the nucleus allowing for the timed activation of MXD3. Here we show that the nuclear translocation of ER-MXD3 initially leads to a transient increase in cell proliferation based on bromodeoxyuridine (BrdU) incorporation but ultimately results in an overall decrease in cell number. Furthermore, we identify candidate MXD3 regulated genes upon acute induction and long-term expression to investigate the opposing activities of MXD3 in the regulation of cellular proliferation in DAOY cells.

## Results and discussion

### ER-MXD3 translocates into the nucleus upon 4-OHT treatment

We have previously shown that MXD3 knock-down reduced proliferation of DAOY medulloblastoma cells, while *persistent* overexpression also decreased cellular proliferation [10]. To distinguish between MXD3’s acute versus long-term effects in DAOY medulloblastoma cells, we developed 4-OHT inducible cell lines that express MXD3 as a fusion to a portion of the mouse estrogen receptor (254 C-terminal amino acid residues, lacking its DNA-binding domain). Furthermore, we have previously found that MXD3 activity is abolished upon mutation of a single amino acid at the 66^th^ position in the basic domain of MXD3 (MXD3.E66D) [10]. The E66D mutation has been shown to disrupt the basic domain binding of other bHLH proteins to the E-box DNA sequence in gel shift assays [23]. Therefore, as a negative control for subsequent experiments, we developed cell lines stably expressing the inducible fusion ER-MXD3.E66D for comparison.

**Figure 1:**
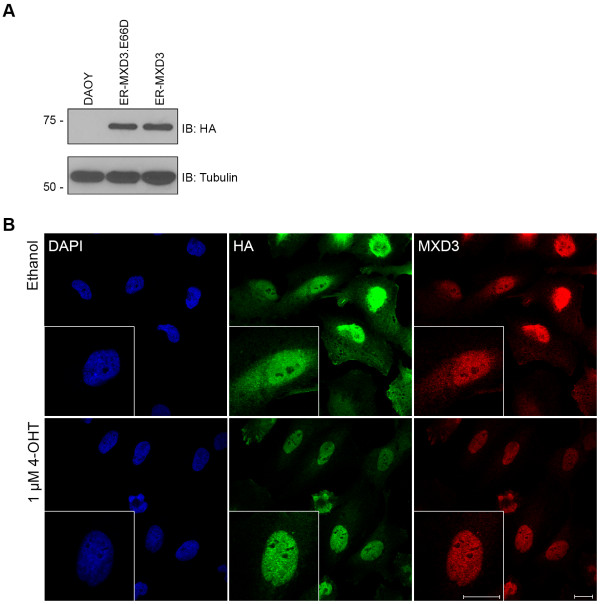
**Characterization of the 4-OHT inducible cell lines. (A)** Extracts from stable cell lines immunoblotted for HA and Tubulin (loading control). DAOY extracts (lane 1) show no expression of HA, while extracts from ER-MXD3.E66D (lane 2) and ER-MXD3 (lane 3) show an expected band of approximately 68 kDa. **(B)** Confocal images of the ER-MXD3 cell line treated with either ethanol (top panels) or 1 μM 4-OHT (bottom panels) for 1 hour and stained for DAPI, HA, and MXD3. When treated with 4-OHT the ER-MXD3 fusion protein is primarily localized in the nucleus (identified by DAPI staining) in contrast to vehicle (ethanol) treatment in which the fusion protein is localized throughout the cell. Scale bar 20 μm.

The fusion protein, ER-MXD3, is expected to be expressed predominantly in the cytoplasm (where it has no known or expected function as a transcription factor) and only translocate to the nucleus (and thus exert its function by binding to target DNA sequences) upon 4-OHT treatment. We confirmed that our cell lines express the respective fusion proteins, ER-MXD3 or ER-MXD3.E66D (Figure 1A and Additional file 1: Figure S1) with no observable degradation or cleavage products (Additional file 2: Figure S2) by immunoblot. Furthermore, the fusion proteins translocate into the nucleus within 1 hour of 4-OHT treatment by immunocytochemistry (Figure 1B). Under vehicle treatment, the fusion protein is present in the cytoplasm as expected but some protein is present in the nucleus (Figure 1B, top panels). This result can be explained by an inherent leakiness within the system or by the presence of trace amounts of an analog of estrogen within the culture media or the cells themselves. We also observe similar results in immunoblotting experiments after biochemical fractionation of nuclear and cytoplasmic components of lysates from our inducible cell lines (Additional file 3: Figure S3). Despite this leakiness, based on the enriched nuclear staining pattern of the fusion protein under 4-OHT conditions (Figure 1B, bottom panels) and the relative localization to the nuclear fraction (Additional file 3: Figure S3), the system can be used for the timed induction of MXD3 transcriptional activity upon nuclear translocation. This timed activation allows for the observation of both immediate and long-term effects of MXD3 activation.

### MXD3 activation results in a transient increase in BrdU incorporation followed by a decrease in cell counts

**Figure 2:**
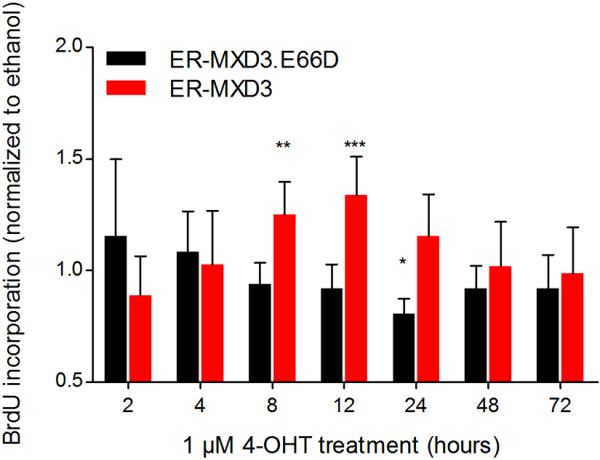
**MXD3 activation induces a transient increase in BrdU incorporation.** Bromodeoxyuridine proliferation assay of ER-MXD3.E66D and ER-MXD3 cell lines treated with 1 μM 4-OHT normalized to vehicle. Treatments were staggered such that all conditions were assayed at the same time for BrdU incorporation after a 2 hour BrdU pulse. ER-MXD3 significantly (p = 0.0487, two-way ANOVA, n = 8) responds to 4-OHT treatment compared to vehicle, whereas ER-MXD3.E66D does not. Results of bonferroni post-test analysis comparing 4-OHT to vehicle are noted with asterisks.

MXD3 has been shown to increase GNP proliferation as measured by BrdU incorporation [9]. To characterize MXD3 in DAOY cells in a time dependent manner, we used our stable cell lines in proliferation assays in which we measured BrdU incorporation in response to MXD3 activation (Figure 2). Two-way ANOVA analysis reveals that there is a significant difference between ethanol vs. 4-OHT treatment in the ER-MXD3 cell line (p = 0.0487) but not in the control ER-MXD3.E66D cell line (p = 0.1595). Bonferroni post-tests show that after 8 hours of 4-OHT treatment there was a significant increase (p < 0.01) in BrdU incorporation in the ER-MXD3 line (Figure 2). This effect peaked at 12 hours post treatment with a significant increase (p < 0.001) of 1.33-fold over vehicle in ER-MXD3. Subsequently, BrdU incorporation returns to baseline (vehicle treated) levels by 72 hours.

**Figure 3:**
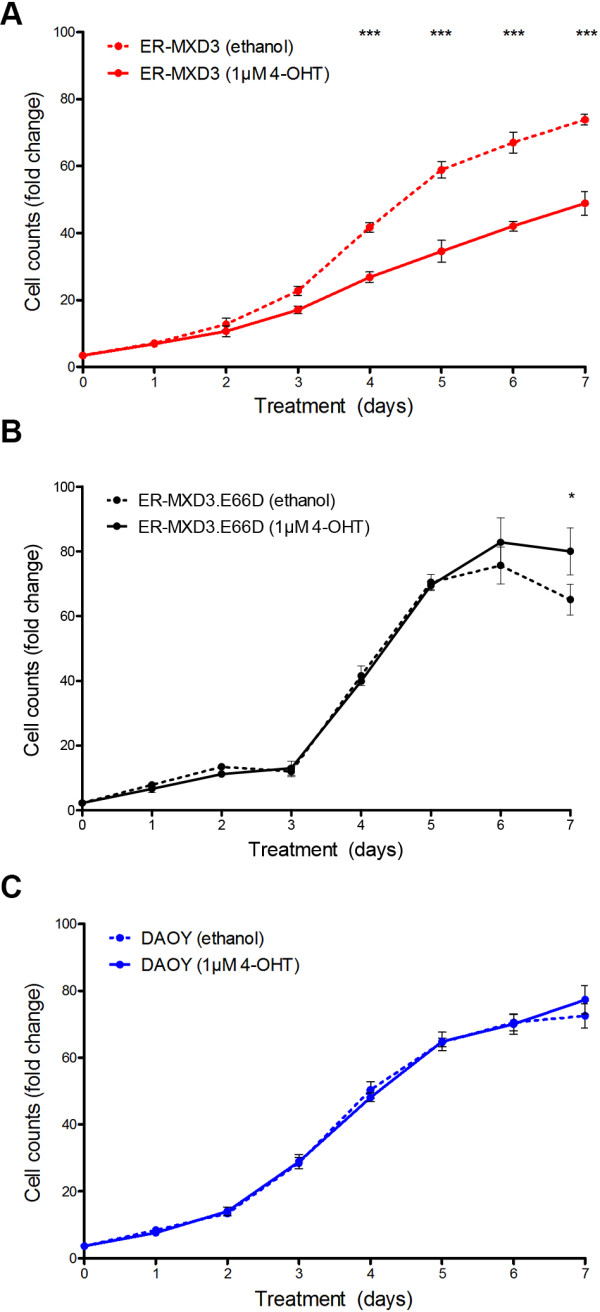
**Persistent MXD3 activation leads to decreased cell counts.** Cell counts of **(A)** ER-MXD3, **(B)** ER-MXD3.E66D, and **(C)** DAOY cell lines over 7 days represented as fold change relative to initial seeding density. ER-MXD3 cell line significantly (p < 0.0001, two-way ANOVA, n = 6) responds to 4-OHT treatment compared to vehicle treatments. Whereas control ER-MXD3.E66D and DAOY lines are unaffected by 4-OHT treatment compared to vehicle treatments. Bonferroni post-test analysis results are noted with asterisks.

As shown in Figure 3 and Additional file 4: Figure S4, however, long-term MXD3 activation results in reduced cell counts, consistent with our previous studies [9,10]. The reduction in cell counts is statistically significant (p < 0.0001) by two-way ANOVA analysis. Bonferroni post-tests reveal that there is a significant difference (p < 0.001) by 4 days post treatment between 4-OHT treated vs. vehicle control (Figure 3A). No significant difference was observed between 4-OHT and vehicle treated in control ER-MXD3.E66D (p = 0.4468) (Figure 3B) and parental DAOY (p = 0.8562) (Figure 3C) cell lines, indicating that this effect is specific to the ER-MXD3 line. Furthermore, these results demonstrate that E-box dependent MXD3 DNA binding is essential for the observed phenotype. ER-MXD3 cells treated with 4-OHT at three times the original seeding density also led to a significant decrease (p < 0.001) in cell number by 48 hours post-treatment (Additional file 5: Figure S5), suggesting that the decrease due to MXD3 activation does not depend on seeding density. On the other hand, withdrawal of 4-OHT after 48 hours failed to significantly rescue cell number (Additional file 5: Figure S5). These results imply that the activation of MXD3 leads to an irreversible decrease in cell number, likely due to the induction of a program of gene expression upon long-term expression of MXD3 (see below).

The MXD3 promoter region has been shown to be regulated by the transcription factor E2F1 [24], a critical transcriptional activator for the transition from the G1 to the S phase of the cell cycle (reviewed in [25]). In agreement, several groups have shown that MXD3 is expressed specifically in the S phase of the cell cycle [9,19,20]. The timing of MXD3 expression suggests that it may play a role in cell cycle progression through the S phase. In support of this possibility, it has been observed that a transient increase in proliferation occurs in both normal mouse GNPs [9] and now in this study of human medulloblastoma cells (Figure 2). The fact that this observation was made in both models suggests that the phenotype observed in response to MXD3 overexpression is a conserved aspect of MXD3 function.

Additionally, the overall decrease in cellular proliferation in response to persistent MXD3 overexpression is also true in both normal [9] and diseased [10] (Figure 3) models. This decrease in proliferation can be explained by the activation of apoptosis as a fail-safe mechanism upon persistent overexpression beyond the S phase of the cell cycle. Such a mechanism exists for oncogenes such as the transcriptional network relative MYC [26,27]. In support of this possibility, both transient overexpression of MXD3 in GNPs [9] and stable MXD3 overexpression in human medulloblastoma cell lines [10] show an increase in apoptosis. When we examined apoptosis activity at 72 hours in our inducible cell lines, we were unable to find any significant difference in caspase 3/7 activity in response to MXD3 activation (Additional file 6: Figure S6); however, there is a trend towards increased caspase 3/7 activity at 72 hours for ER-MXD3, consistent with the decreased cell number observed. Interestingly, the maximal response to hydrogen peroxide treatment (used as a positive control in this assay) in the ER-MXD3 line was 0.608-fold less when compared to the control ER-MXD3.E66D cell line (Additional file 6: Figure S6), suggesting that the capacity of the ER-MXD3 cell line to undergo apoptosis is reduced even in the absence of MXD3 induction. The inherent leakiness in our inducible system could account for this difference between the cell lines. If this is true then it would suggest that at low nuclear concentrations MXD3 functions as an anti-apoptotic factor leading to the subsequent difference in the response to hydrogen peroxide under baseline conditions. Further experimentation will be necessary to test this intriguing possibility.

### Distinct patterns of gene expression are observed upon acute versus long-term MXD3 activation

The results presented thus far indicate that MXD3 has a dual role in DAOY cell proliferation, as we suggested before [10], and that its role is dependent on how long MXD3 is active or present in the nucleus. We report here an initial burst in proliferation (as measured by BrdU incorporation within 12–24 hours) followed by decreased cell counts (at 72 hour) upon MXD3 translocation to the nucleus. Some remaining questions, then, are whether the observed phenotypes due to MXD3 activation are the result of two distinct mechanisms and/or whether the observed phenotypes are the direct result of MXD3 function. These questions remain for both normal and diseased models. To begin to address these questions in the context of human medulloblastoma, we examined the pathways changed in response to MXD3 activation in our inducible cell lines.

To this end, we conducted microarray experiments to compare gene expression between the “early” (increased BrdU incorporation) and “late” (decreased cell counts) effects of MXD3 overexpression. Samples were taken from 12 and 72 hours post treatment from both ER-MXD3 and ER-MXD3.E66D cell lines, in order to define acute and long-term changes in the pattern of gene expression elicited by MXD3. Differentially expressed genes were defined as those genes that showed greater than 2-fold changes in ER-MXD3 (4-OHT/vehicle) over ER-MXD3.E66D (4-OHT/vehicle). It should be noted that this approach aimed at identifying changes that require MXD3 binding to the DNA through its basic domain, as this interaction has been reported to be disrupted in the E66D mutation [10,23,28-30]. A complete list of differentially expressed genes is presented in Additional file 7: Table S1.

**Figure 4:**
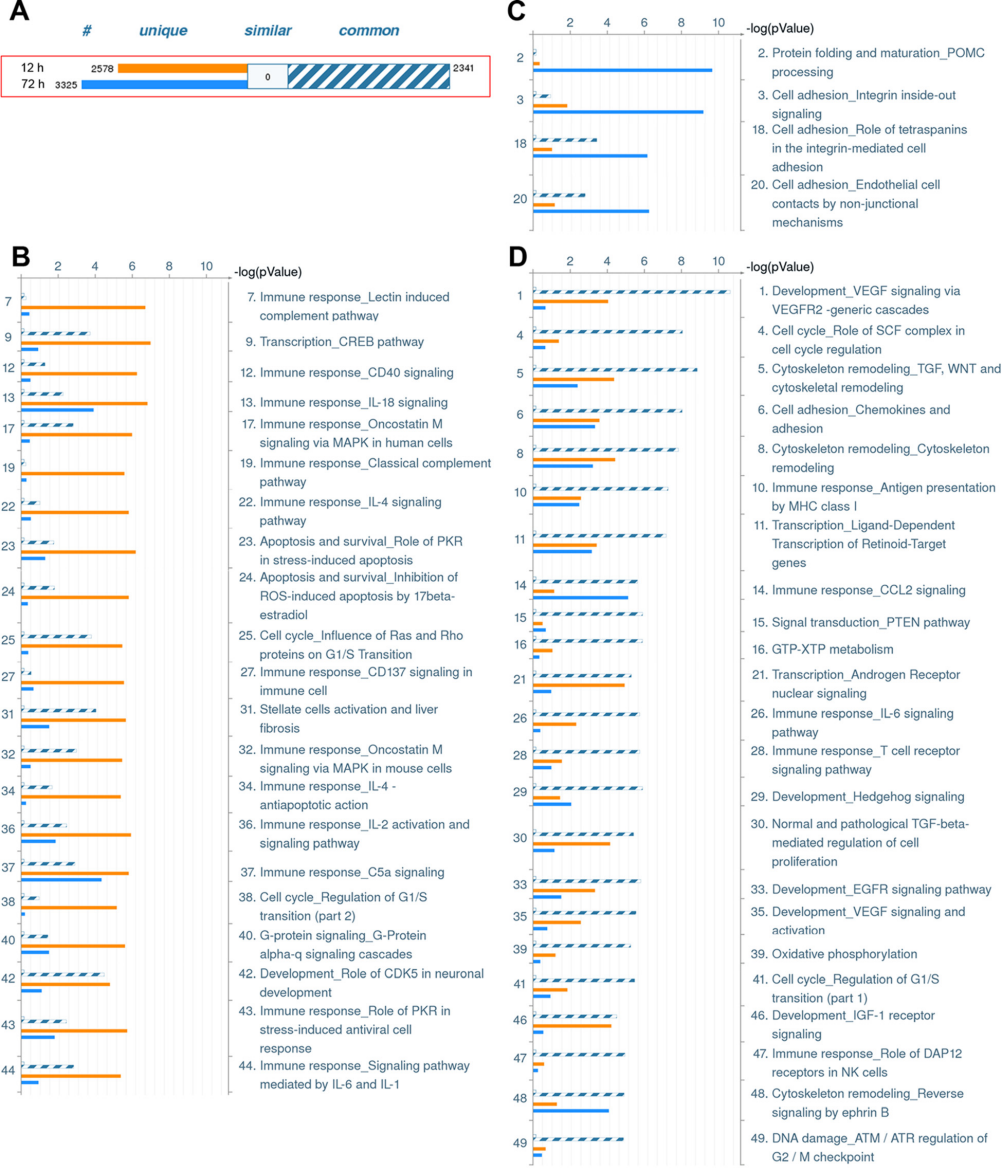
**Gene ontology pathway analysis.** Gene ontology analysis of differentially expressed genes (>2-fold) in ER-MXD3 cells versus ER-E66D cells. **(A)** Overall gene content of the dataset analyzed. Differentially expressed genes at 12 hours (orange bar), 72 hours (blue bar) or both (striped bar) are shown. Canonical pathway enrichment comparison, showing most hits that are more significant for **(B)** 12 hours or **(C)** 72 hours, or **(D)** common to both. Bars colors are as in **(A)**, and their heights represent the p value for a particular set.

**Figure 5:**
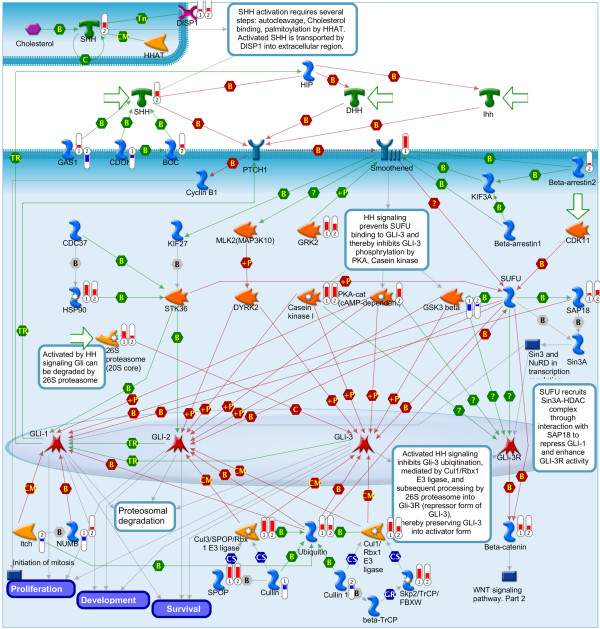
**MXD3 effect on the Hedgehog pathway.** MXD3 activation resulted in differentially expressed gene in the Hh pathway. Thermometer-like icons represent levels of upregulation or downregulation for each specific gene in the 12 hour (➀) or 72 hour (➁) dataset.

Gene ontology analysis of these results is presented in Figure 4. Using MetaCore analysis software, a comparison experiment was performed between differentially expressed genes at 12 hours (“early”) versus 72 hours (“late”). Gene intersection (Figure 4A) showed 2,578 unique early genes (orange bars) and 3,325 unique late genes (blue bars); 2,341 genes were differentially expressed in both time points (striped bars). Pathway comparison analysis is shown in Figures 4B-D. The 50 most differentially affected pathways were grouped according to whether they are mostly represented in the 12-hour data set (Figure 4B) or the 72-hour data set (Figure 4C), or similarly represented in both (Figure 4D). Overall, the 12 hour data set is characterized by pathways related to immune response, apoptosis, and cell cycle regulation. This result is consistent with the proliferative phenotypes we report in this paper; the actual outcome at a specific time is likely the result of the balance between apoptotic and proliferative signals. The immune response pathway might indicate that medulloblastoma cells are undergoing a stress response upon MXD3 activation. The most differentially affected pathways at 72 hours (Figure 4C) include cell adhesion related genes. It can be hypothesized, then, that long term expression of MXD3 results in transcriptional modulation of genes that might explain the observed decrease in cell number due to loss of cell adhesion. The most significantly affected pathways at both time points (Figure 4D) include VEGF signaling, the TGF/WNT pathways, cell cycle regulation, and cytoskeletal remodeling. Interestingly, the Hedgehog (Hh) pathway is also affected. While these pathways are all relevant to several aspects of cancer biology (cell proliferation, migration, metastasis, anchorage independent growth), it is especially important to note the effect of MXD3 overexpression on the Hh pathway, consistent with previous results from our laboratory [9]. Affected genes in or related to the Hh pathway are presented in Figure 5. Interestingly, SHH was found to be upregulated (predominantly at 72 hours), while SMO was upregulated after 12 hours of MXD3 overexpression in the nucleus. Upregulation of SMO could therefore represent an early response leading to Hh mediated proliferation, while subsequent overexpression of SHH could represent an autocrine and/or paracrine proliferative signal. Differentially regulated genes in the cell cycle, VEGF and WNT pathways are presented in Additional file 8: Figures S7, Additional file 9: Figure S8 and Additional file 10: Figure S9. For the complete pathway enrichment analysis, see Additional file 11: Figure S10 and Additional file 12: Table S2. Select target genes from each pathway identified were validated by qRT-PCR (Additional file 13: Table S3).

## Conclusions

In this study, we use 4-OHT inducible human medulloblastoma cell lines to show that MXD3 activation results in a transient increase in cell proliferation. In corroboration with previous studies, persistent activation of MXD3 eventually results in an overall decrease in cell number. With microarray expression profiling we report candidate downstream targets of MXD3 differentially regulated upon acute versus persistent expression of MXD3. Lastly, with gene ontology analysis we identify several major pathways enriched in response to acute (immune response, apoptosis, cell cycle) versus persistent (cell adhesion) MXD3 activation that provide insight into the opposing roles of MXD3 in medulloblastoma proliferation in a time dependent manner.

## Methods

### Constructs

pCMV-HAER (gift from Peggy Farnham) was the backbone vector for the constructs used in this study. HA-MXD3 [10] was subcloned into pCMV-HAER using BamHI restriction sites to produce pCMV-HA-ER-HA-MXD3 (HA = hemagglutinin tag, ER = truncated estrogen receptor). The ER-MXD3.E66D mutant construct was produced using the Quikchange II Site-Directed Mutagenesis Kit (Agilent) according to manufacturer’s instructions. All constructs were verified by sequencing.

### Cell culture

DAOY cells were acquired from ATCC and were cultured in a standard humidified incubator (5% CO_2_, 37°C). Culture media for DAOY (DAOY media) consisted of minimum essential media (Invitrogen) supplemented with 10% Fetal Bovine Serum (Invitrogen), 1 mM Sodium Pyruvate (Invitrogen), 100 μg/ml Penicillin/Streptomycin (Invitrogen). Stable cell lines were maintained with DAOY media supplemented with 800 μg/ml of G418 Sulfate (Cellgro).

### Transfections and stable cell lines

Transfections were performed with Fugene HD transfection reagent (Roche) according to the manufacturer’s instruction. A 5:2 ratio of DNA to transfection reagent was used in the initial transfection for the production of stable cell lines. Briefly, 48 hours after initial seeding, 7 μg of respective constructs was transfected into DAOY cells. Stable cell lines were established using G418 selection (800 μg/ml) 48 hours post-transfection of respective constructs. After one week, cells were passaged to 96-well plates at 1 cell/well. Clones were expanded and subsequently stable expression was confirmed via immunoblotting and immunocytochemistry.

### Antibodies

The following primary antibodies were used in this study: rat anti-HA (Roche), mouse anti-MXD3 (NeuroMab), mouse anti-β-tubulin (Millipore). Secondary antibodies were as follows: goat anti-rat-Cy3 (Jackson ImmunoResearch), donkey anti-mouse-Cy5 (Jackson ImmunoResearch), anti-goat-horse radish peroxidase (HRP) (Vector Labs), and anti-mouse-HRP (MP Biomedical).

### Immunoblotting

Cell extracts were prepared with lysis buffer at pH 7.4 consisting of 150 mM NaCl, 50 mM Tris, 1% Triton-X100, 23.4 μM Leupeptin (Roche), 6.1 μM Aproptinin (Roche), 14.5 μM Pepstatin A (Roche), and 0.1 mM PMSF (Millipore). Protein concentration was determined with a Micro BCA Protein assay kit (Thermo Scientific). Extracts were separated on 12% acrylamide gels under denaturing and reducing conditions and transferred to nitrocellulose membranes. Blots were developed using standard film methods with HRP conjugated secondary antibodies in conjunction with Luminata Crescendo HRP substrate (Millipore).

### Immunocytochemistry

Cells were grown on poly (L)-lysine coated glass coverslips in 6-well plates. Upon collection, cells were washed once with phosphate buffered saline (PBS) and then fixed with 4% paraformaldehyde (Millipore) and permeabilized with 0.01% Triton-X100 in PBS. Coverslips were then incubated overnight at 4°C with primary antibodies in 5% bovine serum albumin in PBS. Secondary antibody incubations were performed in 5% bovine serum albumin in PBS at room temperature for two hours. Subsequently, DAPI staining was performed in PBS at room temperature for 10 minutes. Lastly, coverslips were mounted onto Superfrost Plus microscope slides (Fisher Scientific) using Fluoromount-G (SouthernBiotech) and subsequently imaged with a LSM 710 confocal microscope (Carl Zeiss).

### Proliferation assays

Cells were seeded at 15,000 cells/well in DAOY media supplemented with ethanol (vehicle) at 1:1000; we found that ethanol has an initial positive effect on cellular proliferation (data not shown) and thus we seeded cells in culture media with vehicle in order to account for this initial effect. After 48 hours, cells were treated with 1 μM 4-OHT (Sigma Aldrich) dissolved in ethanol and allowed to proliferate. Cells were counted in triplicate with a Coulter Counter Z1 (Beckman Coulter) after trypsinization. For Bromodeoxyuridine (BrdU) incorporation measurements, BrdU Cell Proliferation Kit (Millipore) was used according to the manufacturer’s instructions with cells seeded at 2×10^3^ cells/well in a 96-well plate. 4-OHT treatment times were staggered such that all samples were processed and assayed simultaneously for BrdU incorporation after a two hour incubation with BrdU.

### Microarrays and data analysis

RNA samples were purified with an RNeasy kit (Qiagen) and quality checked using an Agilent Bioanalyzer. Subsequent reverse transcription and labeling reactions were conducted with Amino Allyl MessageAmp II aRNA Kit (Ambion). cDNA from each time point was collected and labeled with either Cy3 or Cy5 (source). Two control samples were generated by pooling all Cy3 or all Cy5-labeled samples at each time point. Subsequently, samples were hybridized to Whole Human Genome 4×44K microarrays (Agilent) with their associated opposing dye labeled control (e.g. ER-MXD3.12 hours.of.4-OHT.Cy3 + Pool.of.all.samples.Cy5). Microarrays were imaged using an Axon GenePix 4000B microarray scanner (Molecular Devices) and feature extraction was conducted using GenePix Pro 6.0. Fluorescence intensity of features of each sample was normalized to a dye swap pool. Subsequently, 4-OHT time points were normalized to the initial time point or vehicle control producing a ratio of (4-OHT/ethanol). Ratios, then, between ER-MXD3 and ER-MXD3.E66D cell lines were compared to identify MXD3 candidate regulated genes. We defined hits as genes that were up/down-regulated more than two times in ER-MXD3 (4-OHT/ethanol) compared to ER-MXD3.E66D (4-OHT/ethanol). Gene ontology analysis was performed with MetaCore (Thomson Reuters) using the “compare experiments” module with default parameters.

### Statistics

Statistical significance of the BrdU and cell proliferation assays was analyzed via two-way ANOVA with Bonferroni post-tests using GraphPad Prism (GraphPad Software).

### Availability of supporting data

The microarray gene expression dataset supporting the results of this article is available in the Gene Expression Omnibus (GEO) repository, [GSE5903; http://www.ncbi.nlm.nih.gov/geo/query/acc.cgi?acc=GSE58903].

## Abbreviations

BrdU: Bromodeoxyuridine; GNPs: Granule Neuron Precursors; bHLHZ: Basic-helix-loop-helix-leucine-zipper; ER-MXD3: Truncated Estrogen Receptor and full-length MXD3; 4-OHT: Hydroxytamoxifen; Shh: Sonic hedgehog; Hh: Hedgehog.

## Competing interests

The authors declare that they have no competing interest.

## Authors’ contributions

Conceived and designed experiments: TN, GAB, ED. Performed experiments: TN, GAB. Analyzed data: TN, GAB. Provided input and critically analyzed results: ED, KSL. Wrote the manuscript: TN, GAB, ED. All authors read and approved the final manuscript.

## Supplementary Material

Additional file 1: Figure S1 Entire images of the blots in Figure 1 are shown. Click here for file

Additional file 2: Figure S2 Immunoblot of DAOY parental and 4-OHT stable cell lines. HA-ER-HA-MXD3 is expressed as a single fusion protein with no observable degradation or cleavage products detected by immunoblot. Click here for file

Additional file 3: Figure S3 Nuclear and cytosolic fractionation of cell lysates from control ER-MXD3.E66D and experimental ER-MXD3 lines at different time points of tamoxifen treatment. Immunoblotting for HA shows that the fusion proteins disappear from the cytosolic fraction and subsequently become enriched in the nuclear fraction upon tamoxifen treatment. GAPDH was used as a loading control and marker of the cytosolic fraction; histone H3 was used as a loading control and marker of the nuclear fraction. Click here for file

Additional file 4: Figure S4 (A-C) Raw cell counts from Figure 3 are shown. (D) Raw cell counts of the three cell lines treated with vehicle control (ethanol) are shown on the same graph. Click here for file

Additional file 5: Figure S5 (A) Cell counts of ER-MXD3 cell lines treated with 1 μM 4-OHT over 4 days represented as fold change relative to initial cell counts 24 hours after seeding. (B) At 48 hours after vehicle treatment, a subset of Ethanol treated cells were subsequently treated with 1 μM 4-OHT. There was a significant difference between ethanol and the newly treated 4-OHT cells after 48 hours. (C) At 48 hours after tamoxifen treatment, 4-OHT was withdrawn from a subset of cells. There was no significant change upon 4-OHT withdrawal after 48 hours. (D) Graph depicts results from (A-C). Click here for file

Additional file 6: Figure S6 Caspase 3/7 activity of control ER-E66D and experimental ER-MXD3 cell lines treated with either vehicle, 12 hours of 1 μM 4-OHT, 72 hours of 1 μM 4-OHT, or 6 hours of 150 μM H_2_O_2_. Treatments were staggered such that all samples were collected at the same time. Caspase 3/7 activity was measured with a Caspase-Glo 3/7 assay kit (Promega) in a 100 μl reaction volume. Specifically, 50 μl of the caspase detection reagent was substituted with 2x10^3^ cells in 50 μl of media and incubated for 1 hour in a 96-well white walled/clear bottom plate. Subsequently, luminescence was detected using a M5 SpectraMax plate reader (Molecular Devices). Click here for file

Additional file 7: Table S1 Gene expression profiling of ER-MXD3 cell line upon treatment with tamoxifen for 12 or 72 hours. Data represent the fluorescence intensity value normalized to vehicle control. Two-fold changes in gene expression between ER-MXD3 versus ER-E66D were considered to be differentially expressed upon MXD3 translocation to the nucleus. Click here for file

Additional file 8: Figure S7 Role of SCF complex in cell cycle regulation. Pathway was generated with MetaCore analysis software. MXD3 activation resulted in differentially expressed gene in the pathway. Thermometer-like icons represent levels of upregulation or downregulation for each specific gene in the 12 hour (➀) or 72 hour (➁) dataset. Click here for file

Additional file 9: Figure S8 VEGF signaling via VEGFR2, generic cascades. Pathway was generated with MetaCore analysis software. MXD3 activation resulted in differentially expressed gene in the pathway. Thermometer-like icons represent levels of upregulation or downregulation for each specific gene in the 12 hour (➀) or 72 hour (➁) dataset. Click here for file

Additional file 10: Figure S9 WNT signaling. Pathway was generated with MetaCore analysis software. MXD3 activation resulted in differentially expressed gene in the pathway. Thermometer-like icons represent levels of upregulation or downregulation for each specific gene in the 12 hour (➀) or 72 hour (➁) dataset. Click here for file

Additional file 11: Figure S10 Pathway maps enrichment analysis, sorted by differentially affected pathways. Analysis was performed with MetaCore analysis software using default parameters. Click here for file

Additional file 12: Table S2 Pathway enrichment analysis for differentially expressed genes upon MXD3 activation for 12 or 72 hours. For each pathway map, the specific genes found to be differentially expressed are indicated for the common and unique subsets, together with p value and false discovery rate (FDR). Click here for file

Additional file 13: Table S3 Validation of select targets from pathways identified in gene ontology analysis with SYBR green qRT-PCR. Values for each target gene tested are reported as signal normalized to the vehicle control and to the E66D control as follows: (MXD3-4-OHT/MXD3-Vehicle)/(E66D-4-OHT/E66D-Vehicle). Click here for file
