# Supplementary figures and images for: MXD3 regulation of DAOY cell proliferation dictated by time course of activation
Source: BMC Cell Biol. 2014 Jul 23;15:30. doi: 10.1186/1471-2121-15-30 (PMC4226952; doi:10.1186/1471-2121-15-30)

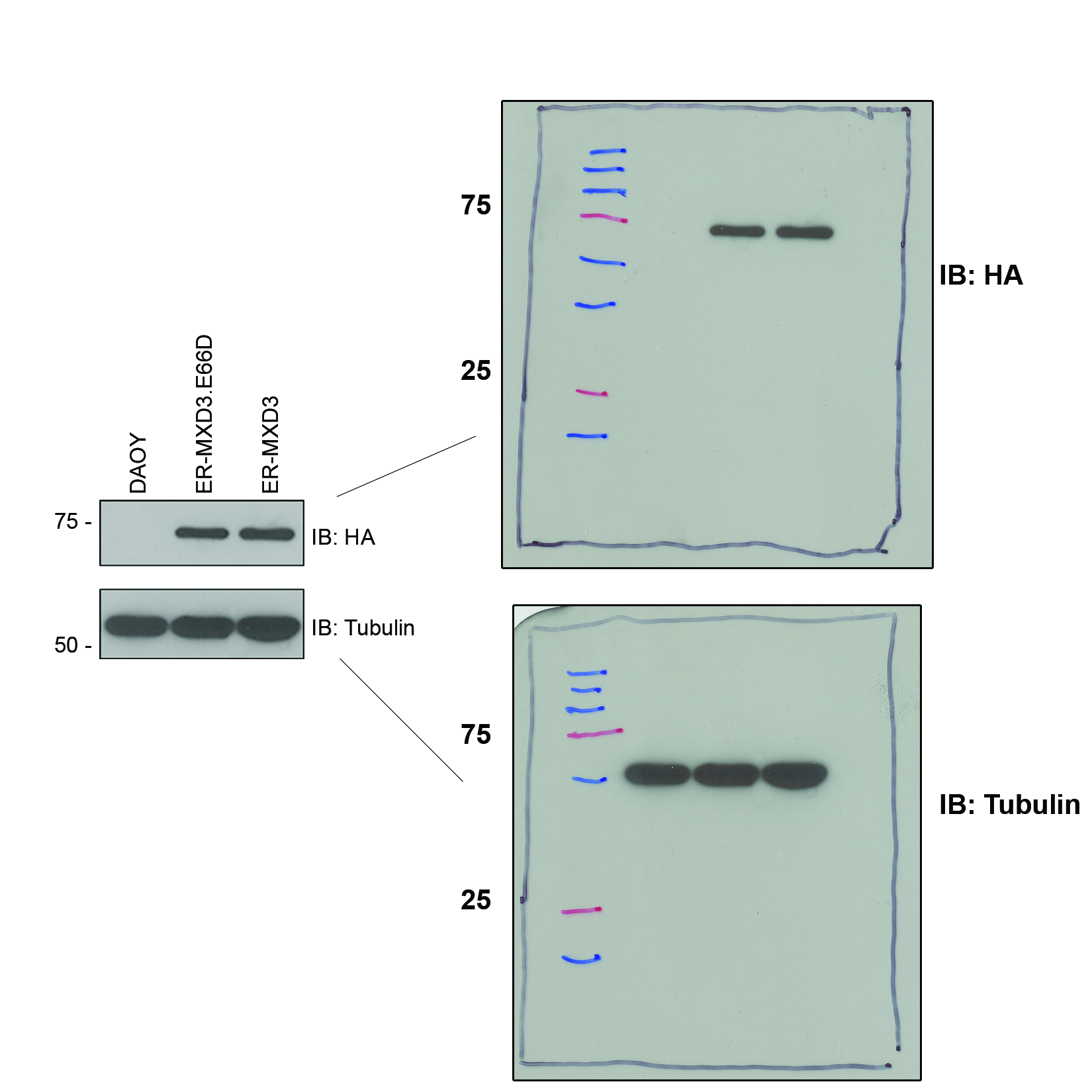

Supplement: Additional file 1: Figure S1 — Entire images of the blots in Figure 1 are shown. [file 1471-2121-15-30-S1.tiff]

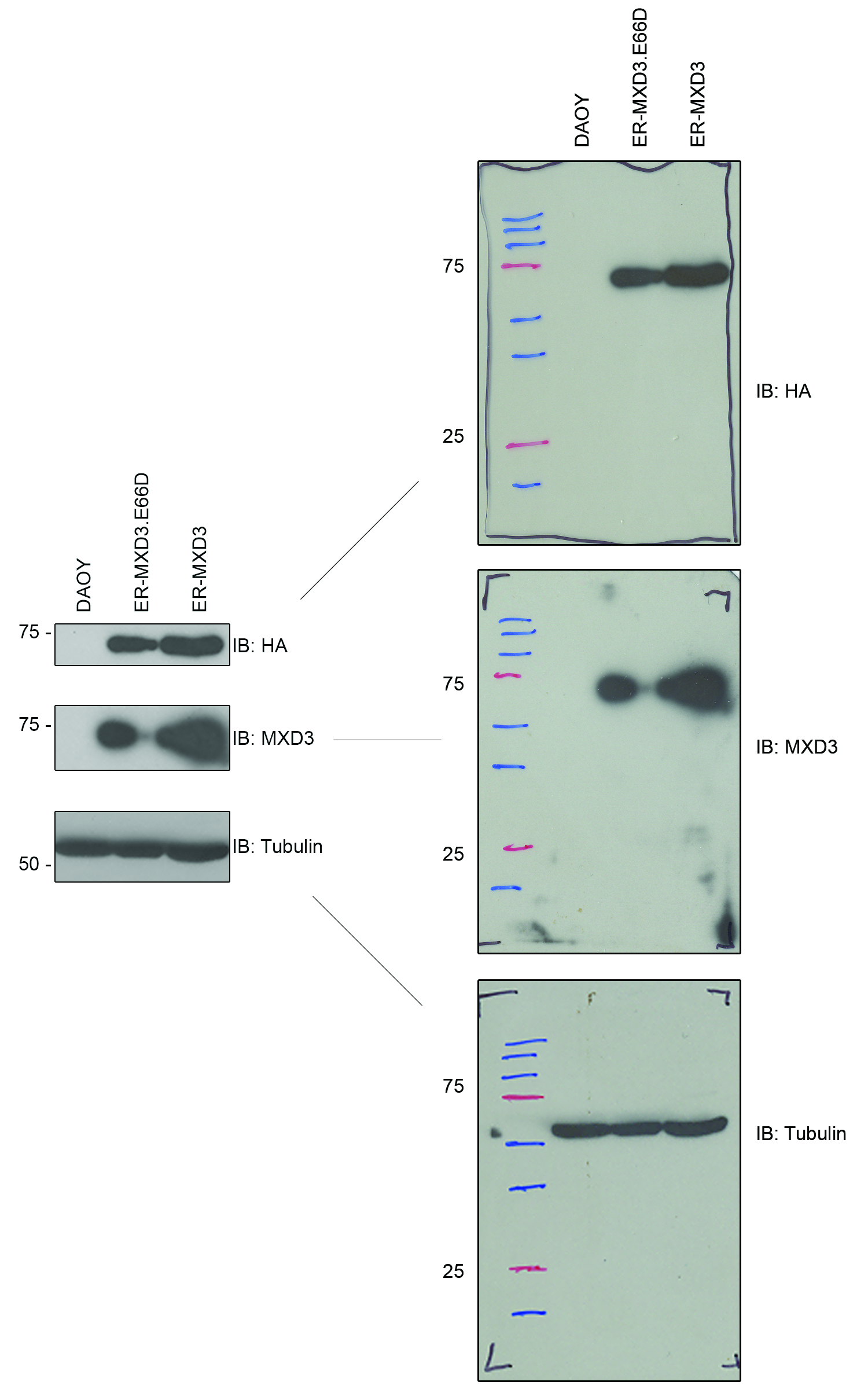

Supplement: Additional file 2: Figure S2 — Immunoblot of DAOY parental and 4-OHT stable cell lines. HA-ER-HA-MXD3 is expressed as a single fusion protein with no observable degradation or cleavage products detected by immunoblot. [file 1471-2121-15-30-S2.tiff]

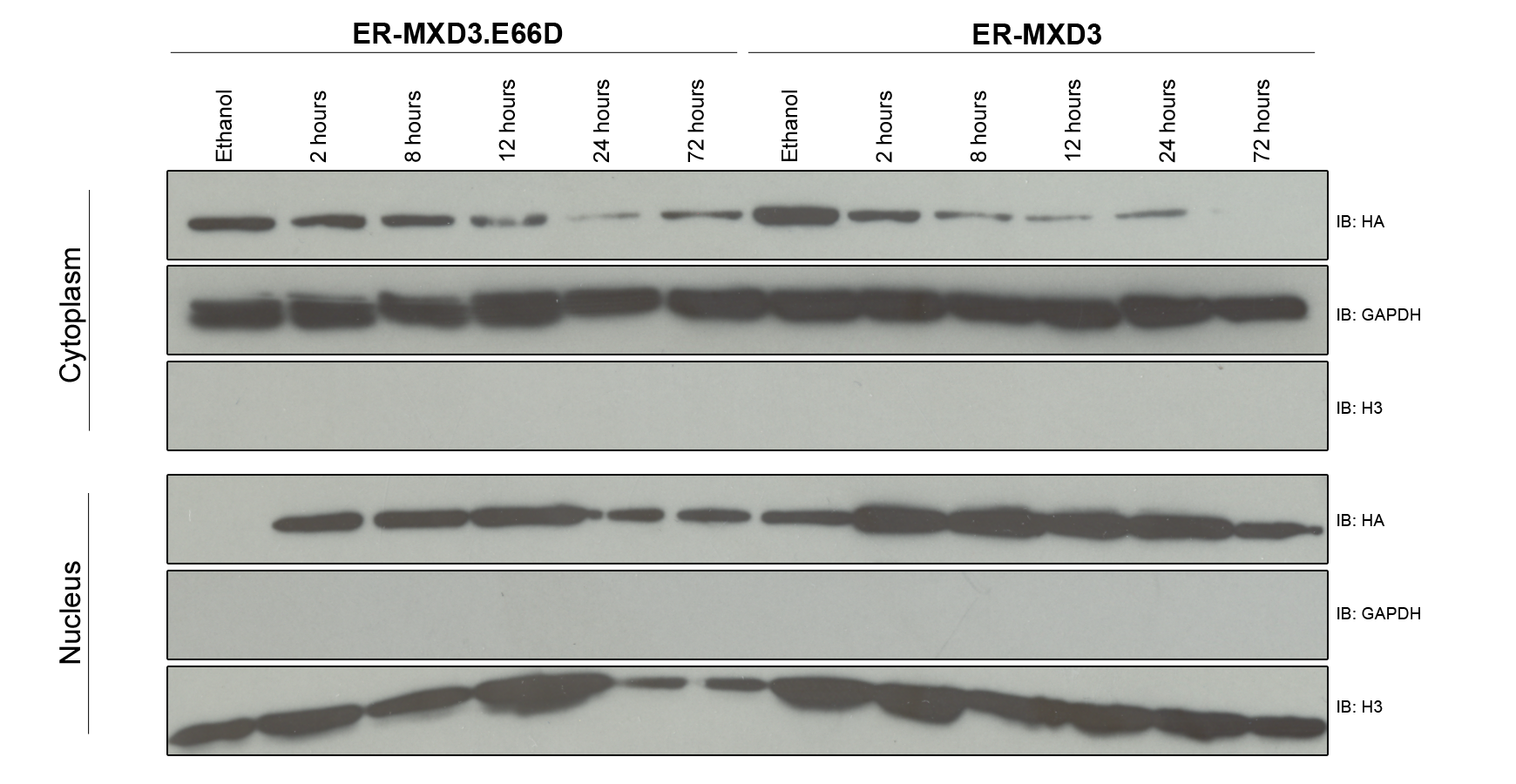

Supplement: Additional file 3: Figure S3 — Nuclear and cytosolic fractionation of cell lysates from control ER-MXD3.E66D and experimental ER-MXD3 lines at different time points of tamoxifen treatment. Immunoblotting for HA shows that the fusion proteins disappear from the cytosolic fraction and subsequently become enriched in the nuclear fraction upon tamoxifen treatment. GAPDH was used as a loading control and marker of the cytosolic fraction; histone H3 was used as a loading control and marker of the nuclear fraction. [file 1471-2121-15-30-S3.tiff]

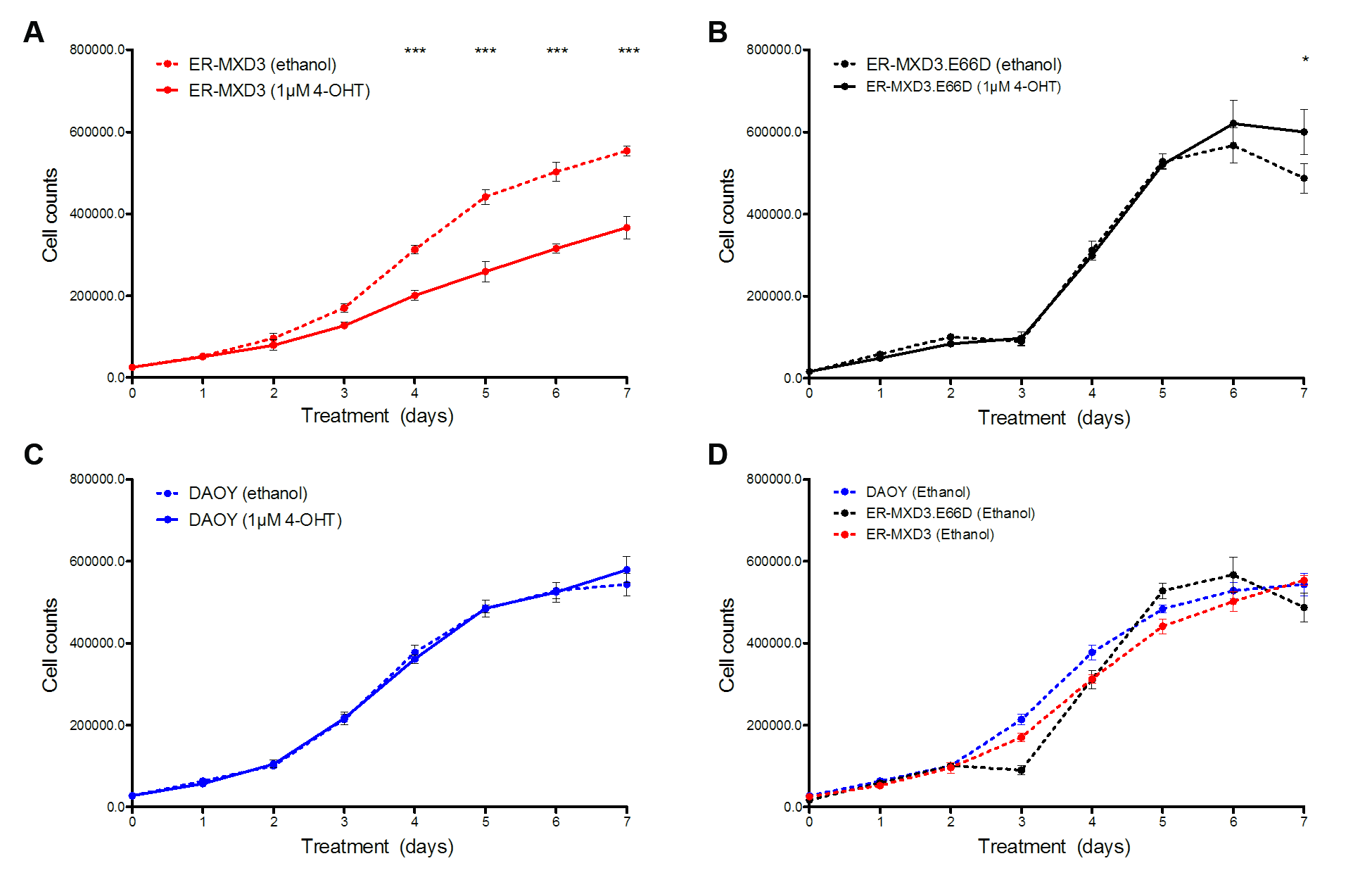

Supplement: Additional file 4: Figure S4 — (A-C) Raw cell counts from Figure 3 are shown. (D) Raw cell counts of the three cell lines treated with vehicle control (ethanol) are shown on the same graph. [file 1471-2121-15-30-S4.tiff]

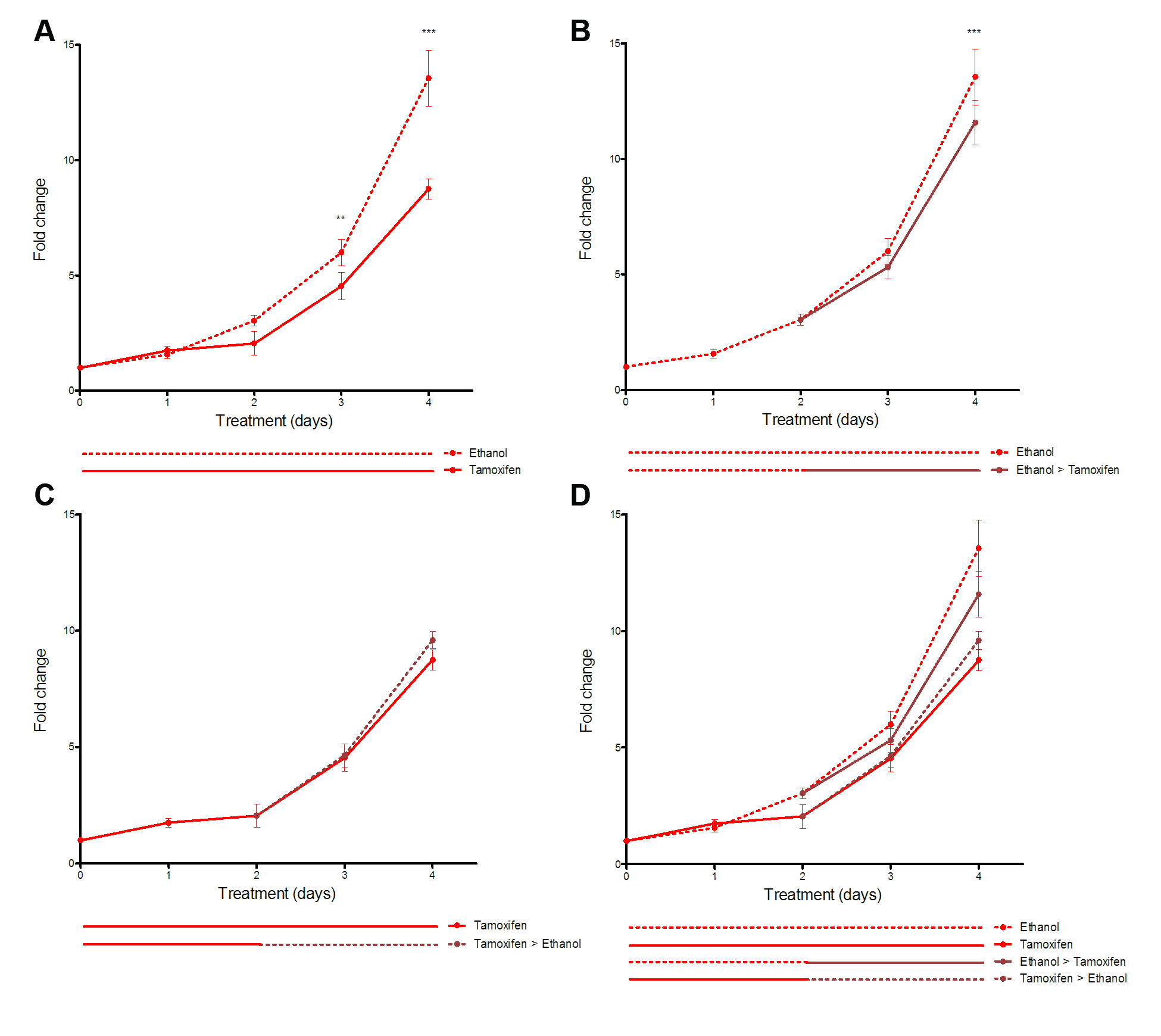

Supplement: Additional file 5: Figure S5 — (A) Cell counts of ER-MXD3 cell lines treated with 1 μM 4-OHT over 4 days represented as fold change relative to initial cell counts 24 hours after seeding. (B) At 48 hours after vehicle treatment, a subset of Ethanol treated cells were subsequently treated with 1 μM 4-OHT. There was a significant difference between ethanol and the newly treated 4-OHT cells after 48 hours. (C) At 48 hours after tamoxifen treatment, 4-OHT was withdrawn from a subset of cells. There was no significant change upon 4-OHT withdrawal after 48 hours. (D) Graph depicts results from (A-C). [file 1471-2121-15-30-S5.tiff]

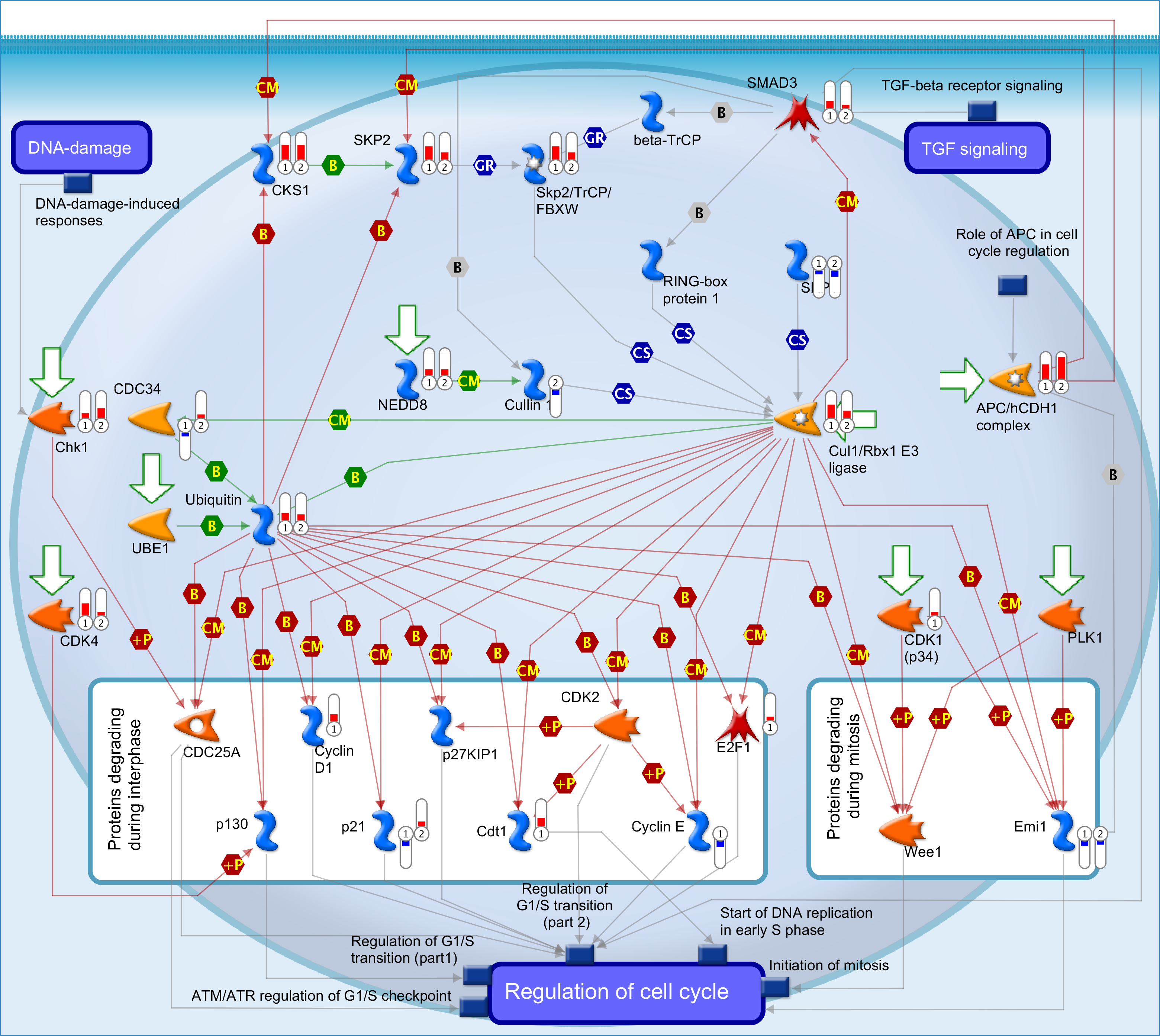

Supplement: Additional file 8: Figure S7 — Role of SCF complex in cell cycle regulation. Pathway was generated with MetaCore analysis software. MXD3 activation resulted in differentially expressed gene in the pathway. Thermometer-like icons represent levels of upregulation or downregulation for each specific gene in the 12 hour (➀) or 72 hour (➁) dataset. [file 1471-2121-15-30-S8.tiff]

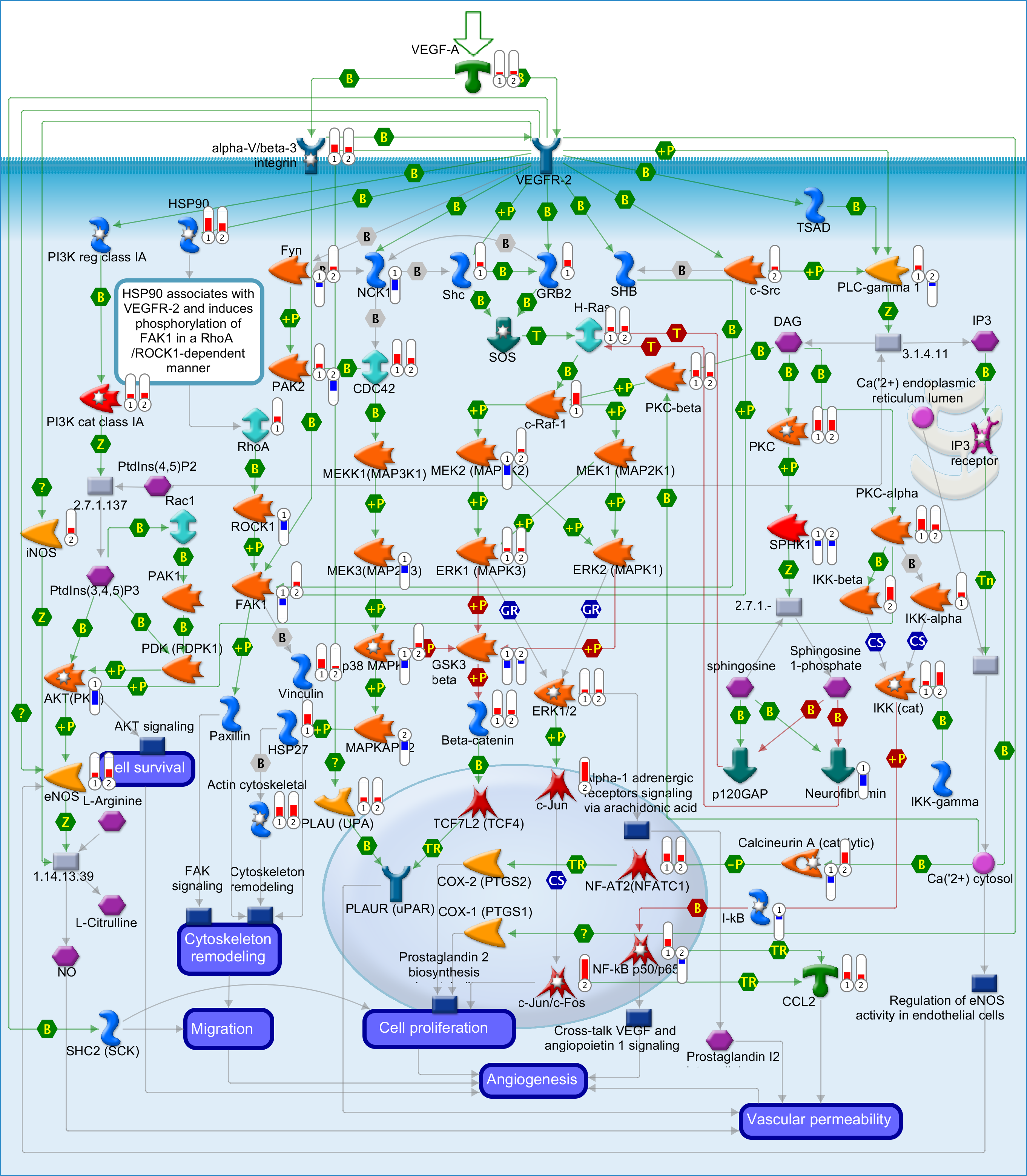

Supplement: Additional file 9: Figure S8 — VEGF signaling via VEGFR2, generic cascades. Pathway was generated with MetaCore analysis software. MXD3 activation resulted in differentially expressed gene in the pathway. Thermometer-like icons represent levels of upregulation or downregulation for each specific gene in the 12 hour (➀) or 72 hour (➁) dataset. [file 1471-2121-15-30-S9.tiff]

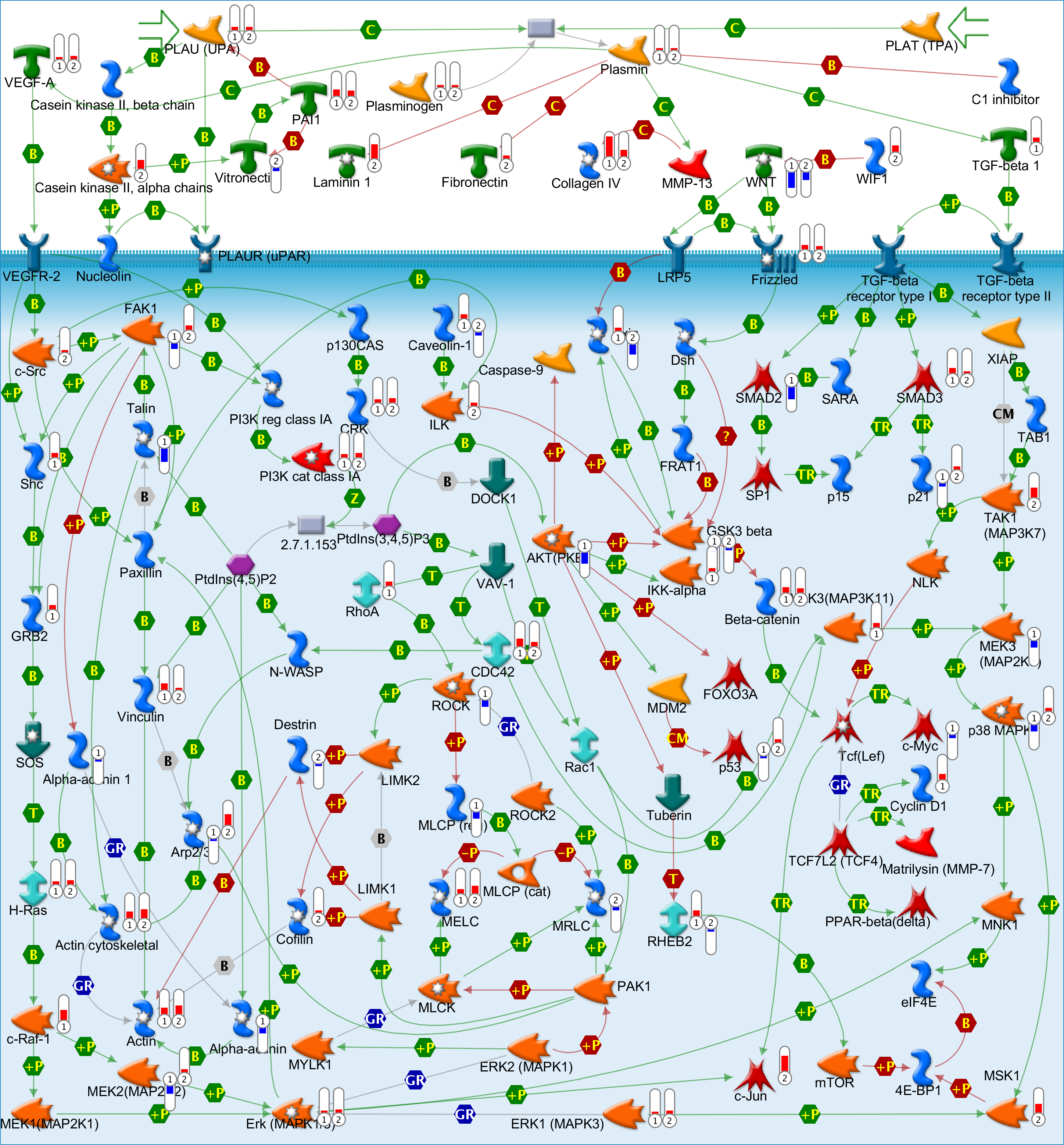

Supplement: Additional file 10: Figure S9 — WNT signaling. Pathway was generated with MetaCore analysis software. MXD3 activation resulted in differentially expressed gene in the pathway. Thermometer-like icons represent levels of upregulation or downregulation for each specific gene in the 12 hour (➀) or 72 hour (➁) dataset. [file 1471-2121-15-30-S10.tiff]

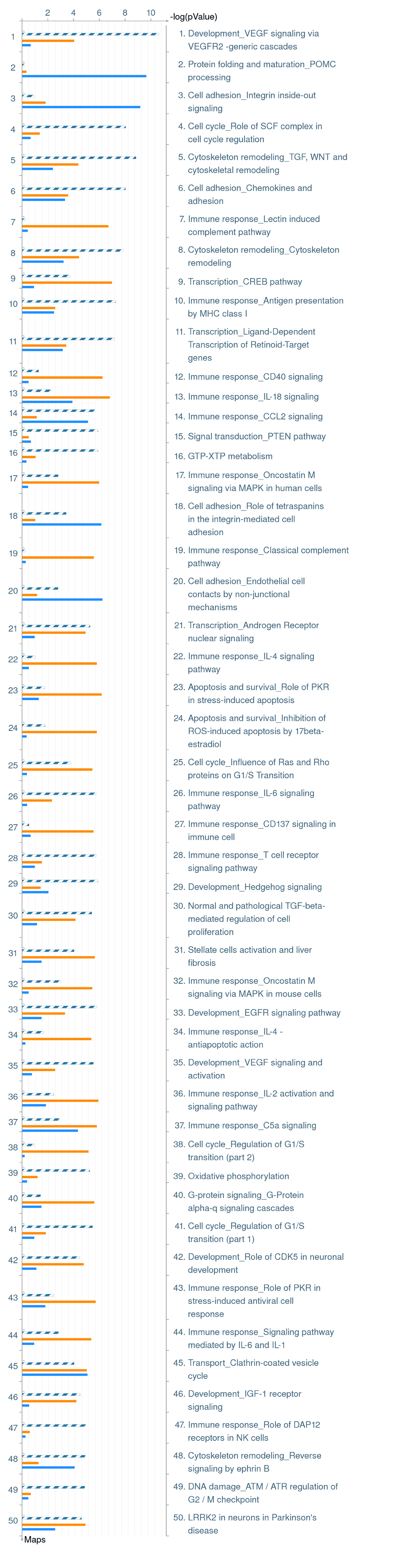

Supplement: Additional file 11: Figure S10 — Pathway maps enrichment analysis, sorted by differentially affected pathways. Analysis was performed with MetaCore analysis software using default parameters. [file 1471-2121-15-30-S11.tiff]
